# The autosomal genetic control of sexually dimorphic traits in humans is largely the same across the sexes

**DOI:** 10.1186/s13059-016-1035-8

**Published:** 2016-08-05

**Authors:** Irfahan Kassam, Allan F. McRae

**Affiliations:** The Queensland Brain Institute, The University of Queensland, St Lucia, QLD 4072 Australia

## Abstract

There are substantial phenotypic differences between the male and female human. Several complex traits have recently been tested to see whether these phenotypic differences are explained by differences in genetic control between males and females. While some differences in genetic control between males and females are detected, overall the results demonstrate that the genetic control of complex traits in humans is largely the same across the sexes.

Please see related Research article: http://genomebiology.biomedcentral.com/articles/10.1186/s13059-016-1025-x

## Introduction

Sexual dimorphism is pervasive in complex traits and diseases, with sex differences observed in, for example, the risk, incidence, prevalence and age-at-onset of cardiovascular disease, cancer, autoimmune disease and neurological and psychiatric disorders [1]. These phenotypic differences between the sexes have often led to the conclusion that the genetic architecture underlying these traits differs between the sexes, with more sexually dimorphic traits having large genetic differences. While genome-wide association studies (GWASs) have been successful at identifying and replicating loci associated with complex traits or diseases in humans, demonstrating a sex-specific genetic architecture of complex traits and diseases has been challenging, and somewhat unsuccessful. Rawlik and colleagues address the question of sex-specific genetic architecture of a range of complex traits by combining the very large sample size provided by the UK Biobank with a complex whole-genome statistical method [2].

## Genome-wide approach to detecting genotype-by-sex interactions

A typical approach to study sex-specific genetic effects has been to perform either a sex-stratified GWAS or genotype-by-sex analysis. With few notable exceptions (e.g. [3, 4]), these analyses are usually secondary to the main analysis in the combined sample, not subject to the same standards of replication and often very underpowered. It has been demonstrated that a study with 80 % power to detect the main effect will only have 29 % power to detect an interaction of the same magnitude in a genotype-by-sex analysis [5]. This has significant implications for the literature for the sex-specific genetic architecture of complex traits and diseases, where the likelihood of false-positive sex-specific genetic effects that are reported is relatively high [6].

Rawlik and colleagues take an alternative approach to detecting genotype-by-sex interaction by treating the genetic architecture of the complex trait on a genome-wide scale separately for males and females, and then estimate the genetic correlation (rG) across the sexes. This approach has been widely used to look at genetic control of sexual dimorphism in a range of animals [7]. The genetic correlation of a complex trait is a measure of the extent to which two traits are influenced by the same genetic variants and takes on values lying between −1 and 1 [8]. However, in practice, where single-nucleotide polymorphism (SNP) microarray data are used, only the tagged causal genetic variants contribute to the genetic correlation estimate. To investigate the sex-specific genetic architecture of a complex trait, we can consider the trait in males and females as two separate traits and estimate the genetic correlation between them.

A genetic correlation of 1 occurs when the genetic control of the complex trait is the same in both sexes. A genetic correlation of less than 1 shows that the genetic control of the trait of interest differs across the sexes and is consistent with a certain amount of sex-specific genetic architecture. In general, the estimates of genetic correlation are subject to large sampling errors and are, therefore, very seldom precise unless performed in very large samples. This approach has previously been applied to height and body mass index (BMI) using a combined sample of *n* = 44,126 unrelated individuals (*n* = 19,323 men and 24,803 women) of European descent from seven GWAS cohorts [9], where no significant sex-specific genetic architecture was found.

## Genetic correlations for 19 complex traits in the UK Biobank

Rawlik and colleagues address the question of sex-specific genetic architecture of human complex traits by examining the genetic correlation of 19 complex traits using 319,038 common autosomal SNPs across 54,040 unrelated males and 59,820 females from the UK Biobank cohort [2]. The analysis of the very large sample across many traits (using the same data collection protocol) requires the use of state-of-the-art statistical methods and makes the estimates of genetic correlation the most precise ever reported in humans. The investigators found that 7 of the 19 complex traits had genetic correlations significantly less than 1 (after correction for multiple testing). These estimates ranged from 0.96 in height to 0.56 for life-time reproductive success (LRS). Unlike a previous study using the same approach, where no significant sex-specific genetic architecture was found when applied to height and BMI [9], here, both height and BMI were shown to have a genetic correlation less than 1. However, the correlation estimates in the two studies were similar, indicating the difference in significance is purely due to the larger sample sizes provided by the UK Biobank. We also need to take care when looking at the lowest of the estimates of genetic correlation. LRS had an estimated genetic correlation of rG = 0.56, but the heritability estimates in males and females were relatively small, making the estimate of genetic correlation less reliable.

**Fig. 1:**
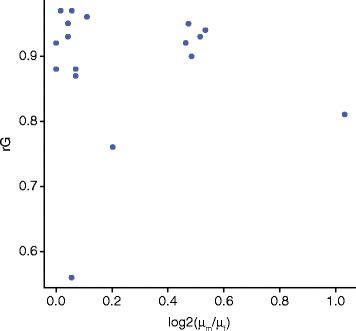
Comparison of estimates of sexual dimorphism and genetic correlation for 19 complex traits. No clear association is observed between the levels of sexual dimorphism for a trait and the genetic correlation between males and females. Derived from Rawlik et al. [2]

There is an assumption when a complex trait has large differences between males and females that these differences are being driven by differences in the genetic control of the trait across the sexes. This assumption appears to be unsubstantiated by the data, with no clear relationship observed between the level of difference between the sexes and their genetic correlation (Fig. 1). In particular, the trait with the lowest genetic correlation has very low phenotypic differences between the sexes, whereas several highly dimorphic traits all have relatively high genetic correlations.

## Concluding remarks

This study demonstrates that most high-level complex traits have very high genetic correlations across the sexes. It follows that individual genotype-by-sex interactions are relatively unimportant, will on average have effect sizes that are much smaller than the main genetic effects on the trait and will thus require very large sample sizes to detect them. On a cautionary note, there is a need for reported genotype-by-sex interaction studies to be held to the high standards already required by GWAS studies, including substantial replication, to prevent the literature being flooded with false-positive results.

## Abbreviations

BMI, body mass index; GWAS, genome-wide association study; LRS, life-time reproductive success; SNP, single-nucleotide polymorphism.
